# Transarterial Chemoembolization of Hepatocellular Carcinoma Using Radiopaque Drug-Eluting Embolics: Impact of Embolic Density and Residual Tumor Perfusion on Tumor Recurrence and Survival

**DOI:** 10.1007/s00270-021-02858-6

**Published:** 2021-05-21

**Authors:** Christer Ruff, Christoph Artzner, Roland Syha, Ulrich Grosse, Rüdiger Hoffmann, Michael Bitzer, Sasan Partovi, Marius Horger, Konstantin Nikolaou, Gerd Grözinger

**Affiliations:** 1 Department of Diagnostic and Interventional Neuroradiology, University Hospital Tuebingen, Hoppe-Seyler-Strasse 3, 72076 Tuebingen, Germany; 2 Department of Diagnostic and Interventional Radiology, University Hospital Tuebingen, Hoppe-Seyler-Strasse 3, 72076 Tuebingen, Germany; 3 Department of Diagnostic and Interventional Radiology, Prosper Hospital, Muehlenstraße 27, 45659 Recklinghausen, Germany; 4 Department of Radiology, Spital Thurgau AG, Waldeggstraße 8A, 8501 Frauenfeld, Switzerland; 5 Department for Internal Medicine I, University Hospital Tuebingen, Otfried-Mueller-Strasse 10, 72076 Tuebingen, Germany; 6 Section of Interventional Radiology, Imaging Institute, Cleveland Clinic Main Campus, Cleveland, OH USA

**Keywords:** Hepatocellular carcinoma (HCC), Transarterial chemoembolization (TACE), Radiopaque drug-eluting embolics (rDEE), C-arm mounted cone-beam CT (CBCT)

## Abstract

**Purpose:**

To evaluate the value of dual-phase parenchymal blood volume (PBV) C-arm mounted cone-beam-CT (CBCT) to enable assessment of radiopaque, doxorubicin-loaded drug-eluting embolics (rDEE) based on the visual degree of embolization, embolic density and residual tumor perfusion as early predictors for tumor recurrence after transarterial chemoembolization (TACE) of hepatocellular carcinoma (HCC).

**Material and Methods:**

Thirty patients (50 HCCs) were prospectively enrolled, underwent cross-sectional imaging before and after TACE using 100–300 µm rDEE and had regular follow-up examinations. Directly before and after the TACE procedure, PBV-CBCT was acquired. The response was evaluated and compared to visual degree of embolization (DE) and embolic density (ED) of rDEE deposits, as well as the presence of residual tumor perfusion (RTP) derived from PBV-CBCT. Outcome was assessed by mid-term tumor response applying mRECIST and patient survival after 12 months.

**Results:**

RTP was detected in 16 HCCs and correlated negatively with DE (*p* = .03*) and ED (*p* = .0009*). The absence of RTP significantly improved lesion-based mid-term response rates regarding complete response (CR, 30/34 (88%) vs 2/16 (12.5%), *p* = .0002*), lesion-based complete response rate was 75% (21/28) for DE ≥ 50% vs. 50% (11/22) for DE < 50% (*p* =  .08) and 82% (27/33) for ED ≥ 2 vs. 29% for ED < 2 (5/17), *p* =  .005*). Thirteen patients were treated with re-TACE within 12 months, 11 of which had shown RTP. 12-month survival rate was 93%.

**Conclusion:**

Residual tumor perfusions as assessed by PBV-CBCT during rDEE-TACE proved to be the best parameter to predict mid-term response.

“Level of Evidence: Level 3”

**Supplementary Information:**

The online version contains supplementary material available at 10.1007/s00270-021-02858-6.

## Introduction

Transarterial chemoembolization (TACE) is an established treatment for intermediate-stage hepatocellular carcinoma (HCC) or as a bridge to liver transplantation (LT) for early-stage HCC [1]. Recently developed radiopaque drug-eluting embolics (rDEE) (DC Bead LUMI™, Boston Scientific plc, Marlborough, MA, the USA) for TACE comprise radiopaque microspheres which enable direct visualization with conventional X-ray imaging [2]. This might aid in more precise delivery of rDEE including superior peri- and post-procedural visualization of their extent and distribution. C-Arm mounted cone-beam CT (CBCT) can be performed during the procedure in the angiography suite for immediate peri-interventional assessment of tumor extent and vascularization [3, 4]. CBCT enhances the detection of tumor feeders and, therefore, gains an increasing importance for TACE guidance [3]. Furthermore, CBCT performed directly after TACE has demonstrated the ability to improve the detection of residual tumor perfusion (RTP) and thus helps to tailor individual treatment [3]. However, due to their inherent opacity, rDEE prohibits the proper visualization of residual tumor after TACE with a single-phase contrast-enhanced CBCT as embolics cannot be distinguished from arterial or parenchymal contrast staining. Therefore, a dual-phase CBCT is pursued with a non-enhanced and a contrast-enhanced phase. This allows the subtraction of the datasets and the calculation of parenchymal blood volume (PBV) and hence measurement of tumor perfusion [3].

An important question arising when using rDEE relates to the possible relationship between their accumulation in HCCs after TACE, the treatment response and, ultimately, patient-centered outcome like tumor recurrence and survival. This parameter already plays a role in predicting the outcome after cTACE with Lipiodol [5]. The implications of peri-procedural evaluation of PBV-CBCT for rDEE-treatment response and outcome have not been elucidated yet. In this prospective study, the extent and localization of rDEE deposit and density as well as RTP were evaluated and correlated with clinical outcomes like tumor response according to the modified Response Evaluation Criteria in Solid Tumors (mRECIST) after 3 months and patient survival at 12 months post- initial rDEE-TACE.

## Materials and Methods

The local institutional ethics committee approved this single-center prospective study. Before enrollment, written informed consent was obtained from all participating subjects.

### Patient Population

Between 06/2017 and 06/2019, 30 patients were prospectively enrolled. Detailed patient data are summarized in Table 1. The study included 25 men (mean age 69.4 ± 8.4 years, range 53–83 years) and five women (mean age 71.1 ± 8.3 years, range 68–83 years) with a total number of 50 HCCs. 13 of these 30 enrolled patients were part of another, previously published study [6]. All patients were classified as stage A or B according to the Barcelona clinic liver cancer (BCLC) classification. The average number of tumor lesions per patient was 1.7 ± 0.9 [range 1–5]. The average lesion diameter was 31.2 ± 20 mm. Inclusion and exclusion criteria for TACE were in line with the CIRSE standards of practice guidelines including blood values contained therein [7]. Criteria were age ≥ 18 years, single or oligo-focal HCC (≤ 5 HCCs), no previous treatment by embolization or ablation therapy, multidisciplinary tumor board decision for TACE treatment and imaging including multiphase CT and MRI, not older than 21 days prior to TACE treatment.

**Table 1:** Baseline characteristics of the 30 enrolled subjects with a total of 50 HCC lesions. BCLC = Barcelona clinic liver cancer classification

Patient	Age	Sex	BCLC classification	Number of lesions	Liver segments	Tumor characteristics
1	66	m	B	2	VIII	encapsulated
					IV	encapsulated
2	64	m	B	2	II/III	infiltrative with portal vein invasion
					VIII	encapsulated
3	72	m	A	1	V/VIII	encapsulated
4	65	m	A	1	II	infiltrative
5	64	m	A	1	II/III	encapsulated
6	83	m	B	1	II/III	encapsulated
7	58	m	B	1	III/IV	encapsulated
8	79	m	A	1	V/VI	encapsulated
9	83	m	B	1	VI/VII/VIII	encapsulated
10	62	m	B	5	V	encapsulated
					IV	encapsulated
11	53	m	A	2	V	encapsulated
					IV	encapsulated
12	62	m	B	1	IV/VIII	encapsulated
13	67	m	A	1	IV	encapsulated
14	71	m	B	2	III	encapsulated
					IV	encapsulated
15	67	m	B	3	VI	encapsulated
					VI	encapsulated
					VIII	encapsulated
16	70	m	B	2	II/IV	encapsulated
					VI	encapsulated
17	54	m	A	1	III	encapsulated
18	80	m	A	1	II	encapsulated
19	65	m	A	2	VI	encapsulated
					VIII	encapsulated
20	80	m	A	1	IV	encapsulated
21	76	m	A	3	IV/VIII	encapsulated
					VII	encapsulated
					VII/VIII	encapsulated
22	76	m	B	1	VI	encapsulated
23	81	m	B	1	VIII	encapsulated
24	69	m	B	2	II	encapsulated
					IV	encapsulated
25	69	m	B	2	VII/VIII	encapsulated
					VIII	encapsulated
26	58	w	A	1	VII/VIIII	encapsulated
27	76	w	B	3	I	encapsulated
					II/III	encapsulated
					VI	encapsulated
28	71	w	A	1	VII/VIII	Infiltrative
29	83	w	B	2	II	encapsulated
					VII	encapsulated
30	68	w	B	2	II/IV	encapsulated
					VIII	encapsulated

### Imaging

All patients received pre-interventional cross-sectional imaging with multiphase CT (volume perfusion CT) including non-enhanced CT scans (SOMATOM Definition Flash, Siemens Healthineers, Forchheim, Germany) and GD-EOB-DTPA-enhanced (Primovist®; Bayer Healthcare, Leverkusen, Germany) multiphase 3 T MRI examinations (Magnetom VIDA, Siemens Healthineers, Erlangen, Germany). Follow-up cross-sectional imaging examinations were performed every 3 months after TACE for a period of 12 months, including multiphase CT or MRI, according to a previously published protocol [6]. Technical details of CT acquisitions are given in Supplemental Table 1.

### Transarterial Chemoembolization

All interventions were performed utilizing the same robotic angiographic suite (Artis Zeego Q, VE 40A, Siemens Healthineers, Forchheim, Germany). Femoral access was used in all cases. Aortography was performed in cases where extrahepatic tumor supply or variant anatomy was suspected. 2.7 or 2.4 Fr microcatheters (Progreat; Terumo, Leuven, Belgium) were used in a 4 Fr catheter for super-selective hepatic angiography and embolization using radiopaque polyvinyl alcohol (PVA) embolic microspheres (DC Bead LUMI™; 100–300 µm; Boston Scientific plc, Marlborough, MA, the USA) loaded with 50 mg doxorubicin. rDEE were reconstituted in pure contrast media (Ultravist 300®, Bayer Healthcare, Leverkusen, Germany) to keep the microspheres in suspension. The administration of microspheres was performed under direct fluoroscopic guidance. TACE was terminated after near stasis in all feeding arteries.

### Dual-Phase Parenchymal Blood Volume C-Arm Mounted Cone-Beam-CT (PBV-CBCT)

Two sets of PBV-CBCT images of the liver were acquired during the TACE procedure. The first dataset was acquired 5 min after hepatic arterial angiography before administering rDEE. The second dataset was acquired after near stasis and stop of the embolization to assess RTP. Each CBCT consisted of an unenhanced phase (“mask run”) and a contrast-enhanced phase (“fill run”). Fill run and mask run were subtracted. A non-rigid registration algorithm was performed to mitigate motion-related differences between the two runs. The arterial input function value is calculated from an automated histogram analysis of the vessel tree and applied as a scaling factor to obtain the actual PBV map [12]. Technical details of image acquisition and post-processing using an automatic reconstruction algorithm, as described previously, are given in Supplemental Table 1 [3, 8, 9].

### Data Analysis

#### Evaluation of Tumor Size, Residual Tumor Perfusion, Embolic Density and Degree of Embolization

**Fig. 1:**
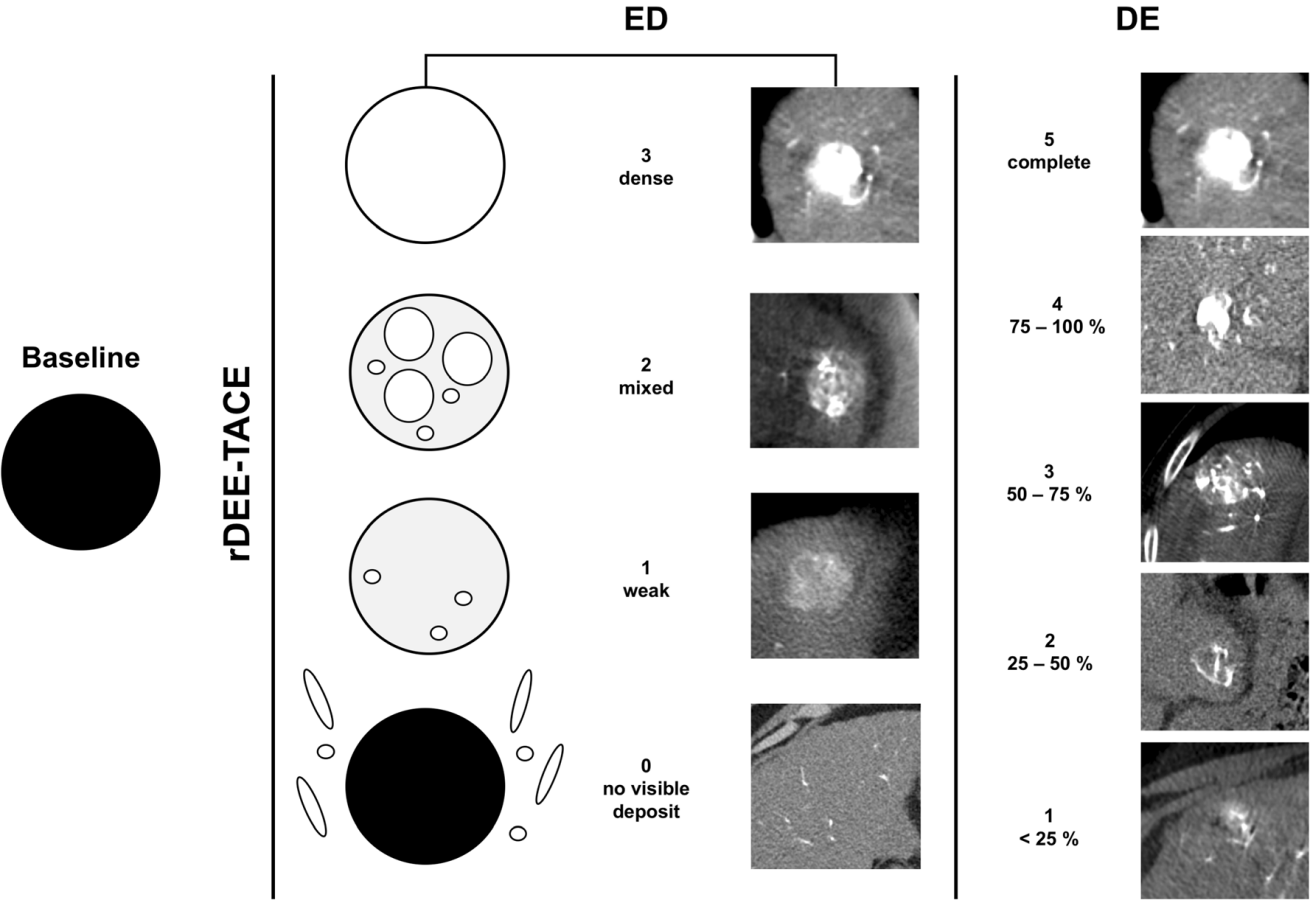
Image examples for the classification of embolic density (ED) and visual degree of embolization (DE). ED and DE (% of the tumor area in cross-sectional images filled with dense radiopaque embolics) of treated HCCs were assessed qualitatively using an ordinal 4-point Likert scale for ED (3 = dense; 2 = mixed; 1 = weak, 0 = not visible) and 6-point Likert scale for DE (5 = complete; 4 = 75–100%; 3 = 50–75%; 2 = 25–50%; 1 =  < 25%, 0 = no visible deposit)

**Fig. 2:**
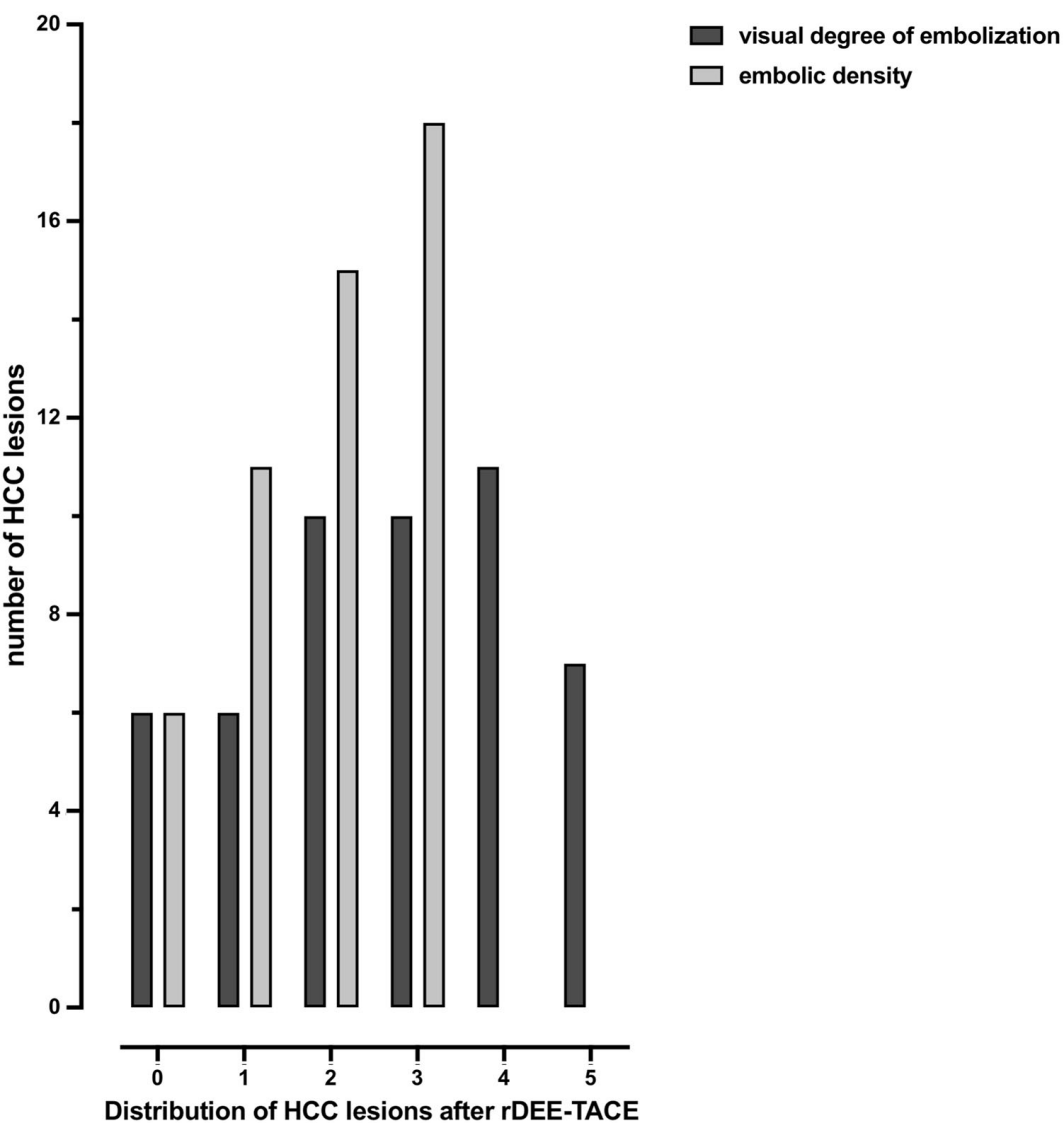
Distribution of HCC lesions according to embolic density (ED) and visual degree of embolization (DE). ED and DE (% of the tumor area in cross-sectional images filled with dense radiopaque embolics) of treated HCCs were assessed qualitatively using an ordinal 4-point Likert scale for ED (3 = dense; 2 = mixed; 1 = weak, 0 = not visible) and 6-point Likert scale for DE (5 = complete; 4 = 75–100%; 3 = 50–75%; 2 = 25–50%; 1 =  < 25%, 0 = no visible deposit). Mean ED was 1.9 ± 1.0 [2, 0-3] and mean DE was 2.7 ± 1.6 [3, 0-5]

In pre-interventional CBCT-PBV maps, maximum lesion diameter was recorded. All post-interventional non-enhanced runs were evaluated for visible contrast media and rDEE deposit. In the case of a present deposit, the maximum diameter of each deposit was measured. Density of rDEE embolization (embolic density, ED) and visual degree of embolization (DE, % of the tumor area in cross-sectional images filled with dense embolic material) of treated HCC lesions were assessed qualitatively using an 4-point Likert scale (3 = dense; 2 = mixed; 1 = weak, 0 = not visible) for ED and a 6-point Likert scale for DE (5 = complete; 4 = 75—100%; 3 = 50—75%; 2 = 25—50%; 1 =  < 25%, 0 = no visible deposit), Fig. 1 and Table 2. ED and DE were evaluated on a representative slice position. Based on the distribution of DE and ED of the HCCs, a categorization of the DE ≥ 50% vs. < 50% and ED of ≥ 2 vs. < 2 was established for further evaluation (Fig. 2, Table 2). On PBV maps, evaluation of residual tumor perfusion of the entire tumor or parts of it was assessed and maximum perfused tumor areas were measured. Two authors (both with 8 and 12 years of experience in cross-sectional imaging and with 4 and 5 years of experience in interventional radiology, respectively) reviewed peri-procedural and follow-up CT, CBCT and MR images in a consensus reading. The readers were blinded to the initial TACE procedure and clinical outcomes including results of mid-term tumor response and 12-month survival.

**Table 2:** Assessment of HCC lesions treated with rDEE-TACE and results of 50 HCC lesions treated with rDEE-TACE according to visual degree of embolization (DE), embolic density (ED) and residual tumor perfusion (RTP)

Likert scale	Meaning	number of HCC lesions
Visual degree of embolization (DE)		
5	Complete	7
4	75—100%	11
3	50—75%	10
2	25—50%	10
1	< 25%	6
0	no visible deposit	6
Embolic density (ED)		
3	Dense	18
2	Mixed	15
1	Weak	11
0	not visible	6
Residual arterial enhancement / tumor perfusion (RTP)		
Yes		16
No		34

An ordinal 4-point Likert scale for ED (3 = dense; 2 = mixed; 1 = weak, 0 = not visible) and a 6-point Likert scale for DE (5 = complete; 4 = 75–100%; 3 = 50–75%; 2 = 25–50%; 1 =  < 25%, 0 = no visible deposit) was used for assessment of rDEE. Mean DE was 2.7 ± 1.6 [1–3, 3–5], and the mean ED was 1.9 ± 1.0 [1, 2, 2, 3]

#### Assessment of Mid-Term Treatment Response

Baseline and 3-month follow-up cross-sectional imaging were evaluated applying mRECIST. Patients were classified as progressive disease (PD, an increase of at least 20% in diameter of the viable lesion), stable disease (SD, any case that does not qualify for either partial response or PD), partial response (PR, at least a 30% decrease in the diameter of the viable lesion), or complete response (CR, the disappearance of any intralesional arterial enhancement). The objective response rate (ORR) was defined as the proportion of patients with CR or PR. Evaluation of tumor recurrence after TACE was performed based on each individual lesion and patient-based.

#### Survival Analysis at 12 Months

Survival analysis was performed at 12 months after the initial TACE. The number of orthotopic liver transplantations (OLT) and the number of repeated TACE procedures were recorded.

### Statistical Analysis

Statistical analysis was performed using JMP 14.2 (SAS Institute Inc., Cary, NC, the USA) and SPSS (release 26 for Windows; SPSS, Chicago, IL, the USA). Median values and interquartile ranges were calculated for the applied ordinal Likert scales. Pearson’s Chi-square test for independence was applied for correlation of DE, ED and RTP. For comparison of all qualitative image parameters of individual patients, Friedman-ANOVA analysis was conducted. If a significant difference was detected, the paired Wilcoxon-signed-rank test was subsequently performed. Due to multiple testing, Bonferroni corrected p values were determined. A corrected p value of equal or less than 0.05 was considered statistically significant.

## Results

### Evaluation of Tumor Size, Residual Tumor Perfusion, Embolic Density and Degree of Embolization

**Fig. 3:**
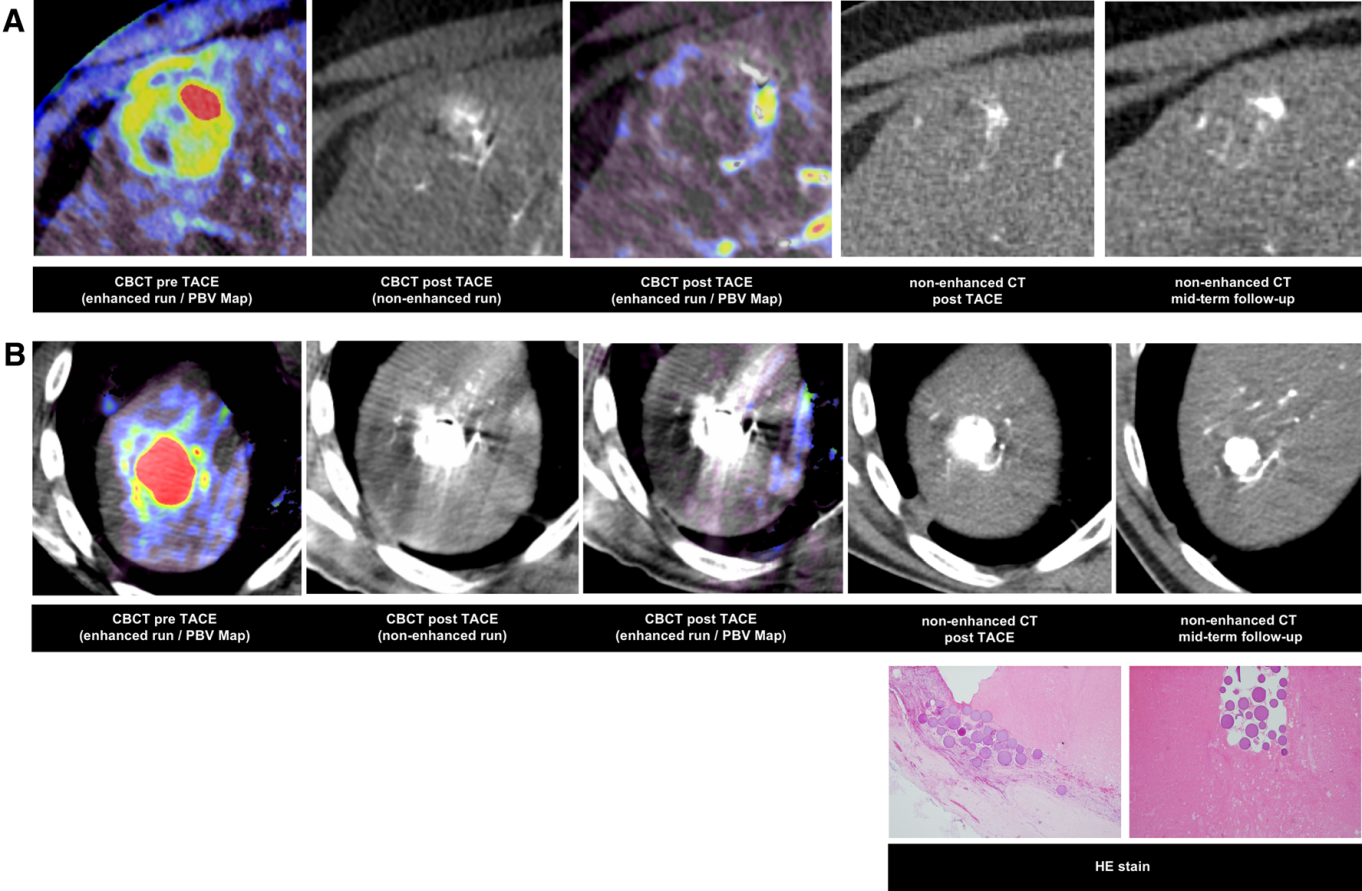
Patient examples with assessment of residual tumor perfusion, embolic density (ED) and degree of embolization (DE) of two patients with HCC lesions post-TACE using radiopaque drug-eluting embolic microspheres (rDEE). **A** A 54-year-old patient with DE of segment III HCC lesion of < 25% and weak ED (1 = weak). PBV map shows residual tumor perfusion (RTP) post-procedurally. **B** A 58-year-old patient with complete embolization (100%) and a high density (3 = dense) of segment VIII HCC lesion post-TACE. In PBV maps, no RTP can be delineated. However, beam hardening artifacts caused by high DC Bead LUMI™ concentration in target lesion is slightly limiting interpretation. The patient received orthotopic liver transplantation 4 months after TACE. Histologic specimen (Hematoxylin and eosin (HE) staining) showed densely packed microspheres within the vessels of the tumor bed surrounded by tumor necrosis

**Fig. 4:**
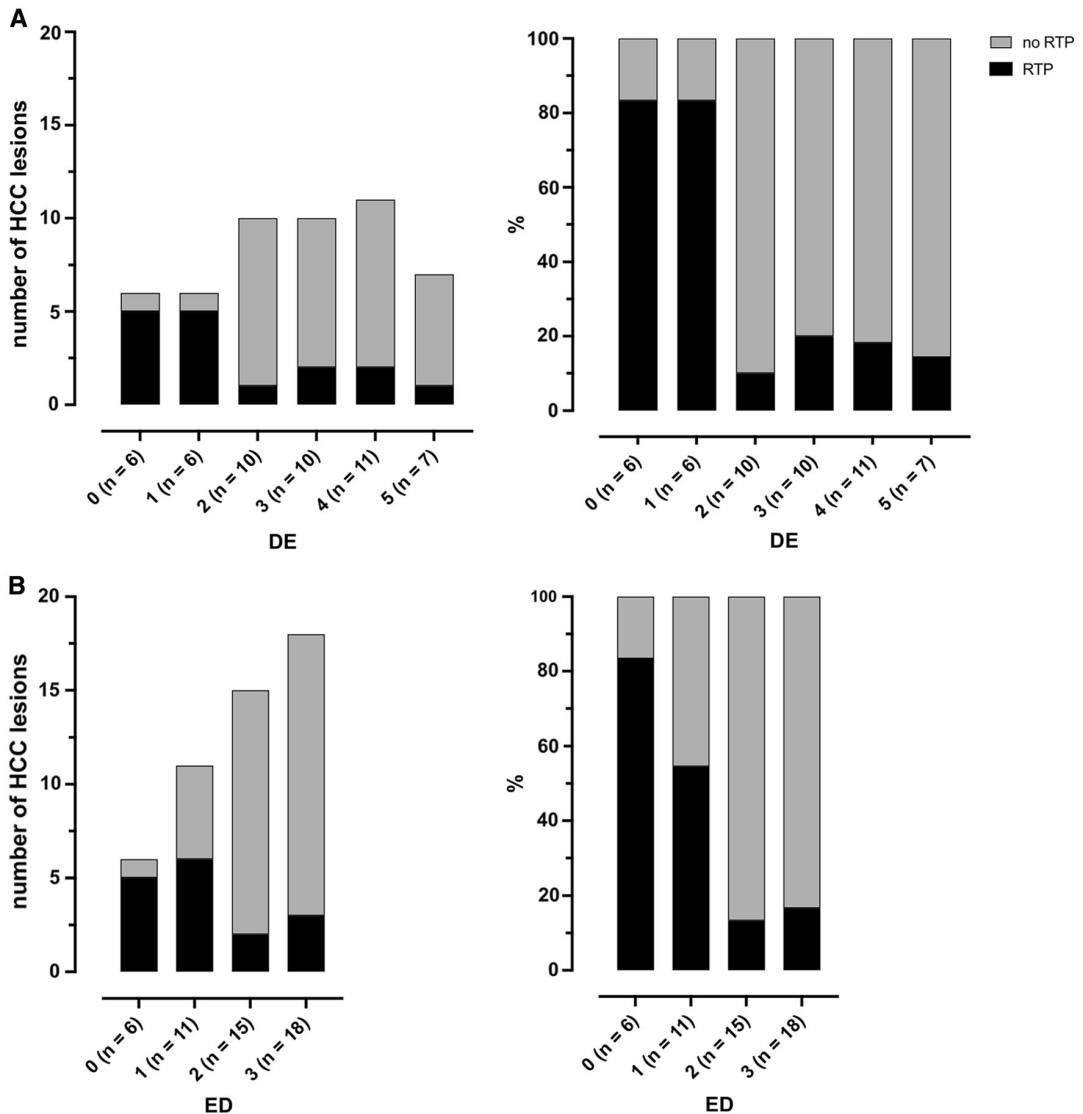
Relationship of residual tumor perfusion (RTP) to degree of embolization (DE, **A**) and embolic density (ED, **B**). Data given in absolute and percentage terms

Three of 50 HCCs (6%) showed a diffuse, infiltrative growth pattern, whereas 47 of 50 HCCs (94%) were encapsulated. The mean size of HCCs in pre-interventional CBCT was 31.2 ± 20 mm. Complete devascularization was achieved in 34 of 50 HCCs (68%). RTP could be detected in 16/50 HCCs (32%). Of the 16 HCCs with RTP, 5 HCCs had a DE ≥ 50% and ED ≥ 2, whereas 11 HCCs had a DE < 50% and ED < 2. RTP was associated with DE < 50% (11/16 (69%), *ρ* = 0.0309*) and ED < 2 (11/16 (69%), *ρ* = 0.0009*), Fig. 3 and 4. There was no significant difference of the size of HCCs with RTP (30.5 ± 11.9 mm) compared to HCCs without detectable RTP (30.8 ± 21.3 mm), *p* =  0.96. The size of detected RTP was 16.1 ± 8.7 mm in CBCT. The largest size of RTP was 26.2 mm.

### Assessment of Mid-Term Treatment Response

**Fig. 5:**
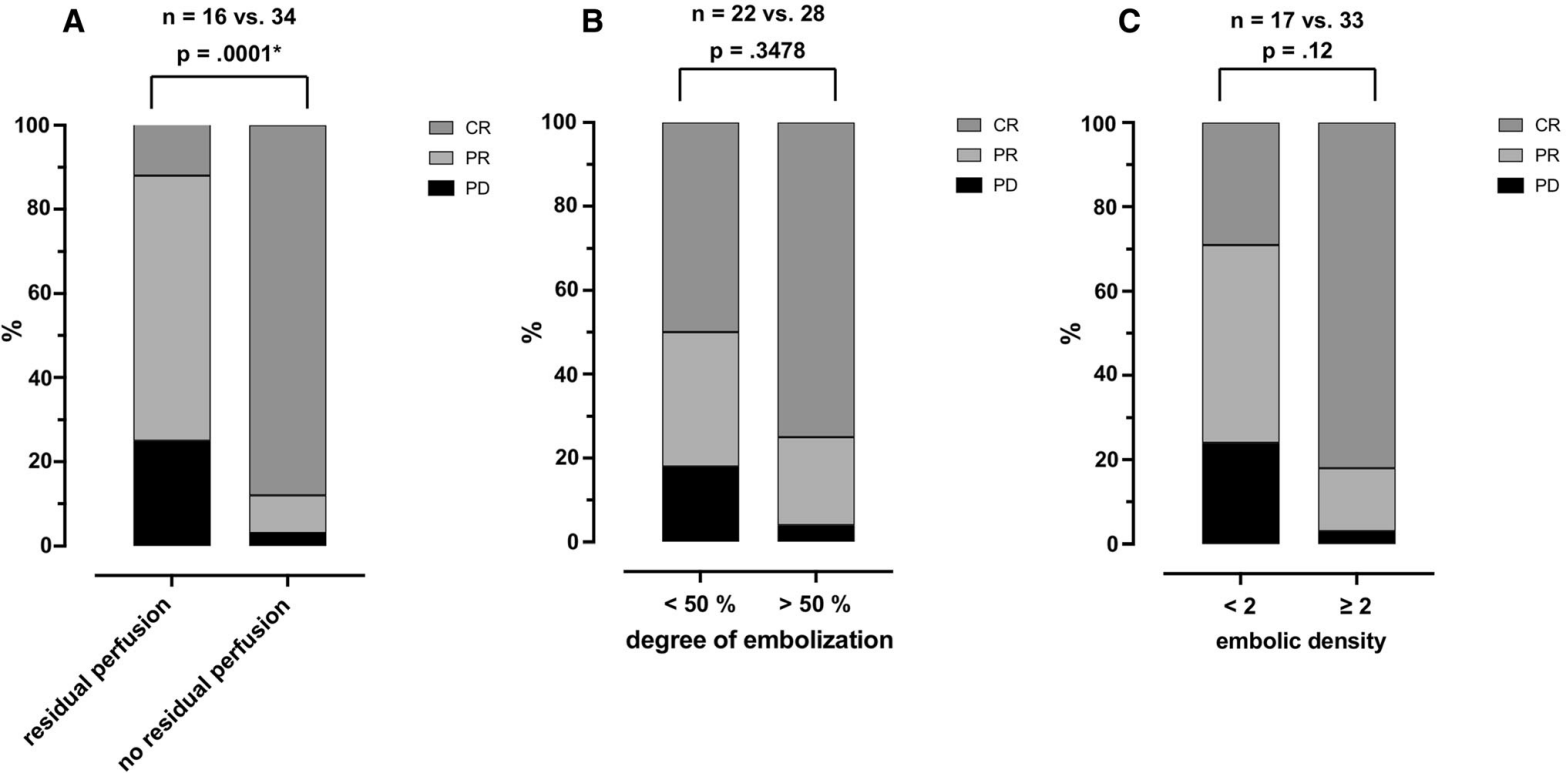
Mid-term response in relation to residual tumor perfusion (RTP, **A**), visual degree of embolization (DE, **B**) ≥ / < 50% and embolic density (ED, **C**) < / ≥ 2 in relationship to complete response (CR), partial response (PR) and progressive disease (PD) based on mRECIST (modified Response Evaluation Criteria in Solid Tumors) criteria. HCC lesions without RTP show superior mid-term tumor response three months post-rDEE-TACE

After 3 months of follow-up, 32/50 HCCs (64%) were classified as CR, 13/50 HCCs (26%) as PR and 5/50 HCCs (10%) as PD according to mRECIST. Figure 5 and Table 3 show the results of RTP, DE and ED in relation to the mid-term response. In addition to the lesion-based response evaluation, a patient- based analysis was performed (Supplemental Table 2). HCCs without RTP had a significant higher level of CR compared to HCCs with RTP (30/34 (88%) vs. 2/16 (12.5%), *p* = 0.0002*). This also resulted in a significantly higher rate of ORR (33/34 (97%) vs 12/16 (75%), *p* = 0.019*). HCCs with DE ≥ 50% had a non-significant trend toward a higher level of CR (21/28 (75%)) compared to HCCs with a DE of < 50% (11/22 (50%), *p* = 0.08. HCCs with ED ≥ 2 had a significantly higher level of CR (27/33 (82%) vs. 5/17 (29%), *p* = 0.005), whereas PR and PD were not significantly different compared to HCCs with ED < 2. ORR, however, was significantly different between these two groups (32/33 (97%) vs. 10/17 (58%), *p* = 0.0062*).

**Table 3:** Results of 50 HCC lesions treated with rDEE-TACE according to visual degree of embolization (DE), embolic density (ED) and residual tumor perfusion (RTP) as well as evaluation based on mRECIST (Modified Response Evaluation Criteria in Solid Tumors) criteria. ORR = objective response rate, CR = complete response, PR = partial response, PD = progressive disease

mRECIST (lesion-based)				
	ORR	CR	PR	PD
Visual degree of embolization (DE)				
< 50	18/22 (82%)	11/22 (50%)	7/22 (31%)	4/22 (18%)
> 50	27/28 (96%)	21/28 (75%)	6/28 (21%)	1/28 (5%)
< 50 vs. > 50	.08	.08	.78	.215
Embolic density (ED)				
< 2	10/17 (58%)	5/17 (29%)	8/17 (47%)	4/17 (24%)
≥ 2	32/33 (97%)	27/33 (82%)	5/33 (15%)	1/33 (3%)
< 2 vs. ≥ 2	.0062*	.005*	.4097	.215
Residual tumor perfusion (RTP)				
Yes	12/16 (75%)	2/16 (12.5%)	4/16 (25%)	10/16 (62.5%)
No	33/34 (97%)	30/34 (88%)	1/34 (3%)	3/34 (9%)
yes vs. no	.019*	.0002*	.0674	.2150

### Survival Analysis at 12 Months

Overall survival at 12 months was 93% (28/30 patients). At 12 months, 9/30 patients had received orthotopic liver transplantation (7.4 ± 3.4 months after the 1^st^ TACE). 13/30 patients had received a second TACE procedure within the first 12 months of follow-up. Of these, 11/13 patients had at least one HCC lesion which had shown RTP in the initial post-TACE PBV-CBCT.

## Discussion

In this study, the value of post-interventional PBV-CBCT could be demonstrated using radiopaque embolics. It enables the assessment of rDEE deposition parameters like embolic density and degree of embolization together with the detection of residual tumor perfusion in treated HCCs. Residual tumor perfusion has proved to be a robust parameter regarding prediction of mRECIST and is influenced by embolic density as well as degree of embolization.

Recently developed rDEE are supposed to improve visibility and lesion targeting [10]. This prospective study, therefore, evaluated CBCT imaging biomarkers in the peri-interventional management of HCCs treated with rDEE-TACE. The aim was to establish early predictors for mid-term tumor response. Non-enhanced CBCT images were evaluated to assess the extent and the visibility of rDEE, whereas PBV-CBCT was used to directly measure residual tumor perfusion after the embolization procedures [3]. Perfusion-based imaging has been shown to accurately assess hypovascularized HCCs as well as the achieved level of tumor devascularization [3, 11–17]. Syha et al. could show that detection of residual tumor perfusion in PBV-CBCT imaging of treated HCCs with drug-eluting embolics was associated with a worse mid-term response. In this study using rDEE, the most robust imaging biomarker also was the absence of residual tumor perfusion with regard to the prediction of mid-term response. The rate of residual tumor perfusion (32%) is in line with previous studies [3, 18]. If possible, detection of residual tumor perfusion should lead to immediate re-treatment. However, immediate treatment with further embolization is not always possible due to the occurence of post-embolization syndrome or impaired liver function with and increasing risk of liver damage.

In this study, rDEE were reconstituted in pure contrast media in all procedures. This is necessary to avoid the immediate sinking of the relatively heavy radiopaque particles. Because of that, the contrast staining within the tumor is certainly iodine-containing contrast medium combined with rDEE. However, this is a constant among all procedures and a dual-phase CBCT enables the discrimination of residual tumor perfusion and staining in the tumor bed. As described in the literature, radiopaque embolics tend to form clots at high concentrations in a model of a phantom study [6]. Furthermore, non-enhanced CT imaging one day after TACE procedure and on mid-term follow-up imaging shows a very similar distribution of the embolics compared to the time of post-interventional CBCT. Low embolic density and degree of embolization were specific findings that were closely linked to the presence of residual tumor perfusion. As such, these parameters may play a complementary role as outcome predictors, even when no dual-phase CBCT with the ability to create PBV maps is available. In these cases, the deposit of rDEE can also be visualized in a separate non-enhanced CT scan one day after rDEE-TACE. A decreased embolic density was significantly associated with an unfavorable mid-term outcome in the form of a reduced rate of complete response. These parameters were already established to predict the outcome after conventional TACE (cTACE) with Lipiodol [5]. In cases with residual tumor perfusion and low embolic density, short re-treatment intervals versus the combination with other local or systemic therapeutic strategies (e.g., radiofrequency ablation) are strategies to improve the individual patient outcome. This is in line with a previous study showing that shorter re-treatment intervals (e.g., six versus 12 weeks) could improve the outcome in cases with residual tumor perfusion after the first TACE session [8].

rDEE distribution and density might pave the way for a “rDEE dosimetry.” This is supported by the results of Sharma et al., who demonstrated that rDEE concentration could be correlated with CT attenuation. Subsequently, the degree of radiopacity post-TACE can be correlated with the delivered drug concentration to the HCC lesions (19). Although rDEE dosimetry was not directly applied in this study, it was demonstrated that embolic density and degree of embolization are linked to the established imaging biomarker of residual tumor perfusion and, thus, might help to estimate the tumor response.

The study has several limitations. The study population consists of a comparably small sample size and the follow-up period was limited to 12 months. Nevertheless, the study results point to a potential path toward a more individualized oncological strategy.

### Conclusion

In conclusion, the results of this study demonstrate the value of post-interventional PBV-CBCT to enable assessment of rDEE deposition parameters like embolic density and visual degree of density together with the detection of residual tumor perfusion in treated HCCs. Residual tumor perfusion has proved to be a robust parameter regarding mid-term response according to mRECIST.

## Supplementary Information

Below is the link to the electronic supplementary material.

Supplementary file1 (DOCX 25 KB)
