# Supplementary material for: Transarterial Chemoembolization of Hepatocellular Carcinoma Using Radiopaque Drug-Eluting Embolics: Impact of Embolic Density and Residual Tumor Perfusion on Tumor Recurrence and Survival
Source: Cardiovasc Intervent Radiol. 2021 May 21;44(9):1403–13. doi: 10.1007/s00270-021-02858-6 (PMC8382629; doi:10.1007/s00270-021-02858-6)
Supplement: Supplementary file 1 — Supplementary file1 (DOCX 25 KB) [file 270_2021_2858_MOESM1_ESM.docx]

|  | VPCT | CBCT |
| --- | --- | --- |
| Examination time [s] | 40 | 16 |
| time per rotation [s] | 1.5 | 4 |
| tube current and tube voltage | 80 kVp, 100 mAs | automatically adjusted |
| Contrast agent | Ultravist 370 | Ultravist 300 |
| Amount of contrast media | 50 to 60 ml | 7.5 ml diluted  to 30 ml |
| Flow rate | 5 ml/s | 3 ml/s |

**Supplemental Table 1.** Acquisition parameter of volume perfusion computed tomography (VPCT) and C-arm mounted computed-tomography (CBCT) used in this study. Collimation of VPCT was 64 × 0.6 mm. In VPCT axial images with a slice thickness of 3 mm for perfusion analysis were reconstructed with a medium smooth tissue convolution kernel (B10f) and transferred to an external workstation (Multi-Modality Workplace, Siemens Healthineers, Forchheim, Germany) for dedicated analysis. In CBCT 30 ml diluted contrast medium was administered followed by a 22.5 ml normal saline chaser. This was injected after the mask run by an automated power injector (Accutron-HP-D, Medtron AG, Saarbrücken, Germany) using a flow rate of 3 ml/s. Acquisition parameters of CBCT were 200° total angle, 0.8° per frame, 248 frames, matrix size 616x480 and pixel size 616 µm. Image reconstruction of CBCT was conducted on a multimodality workstation (MMWP VD 10, Siemens Healthineers, Forchheim, Germany). A non-rigid registration algorithm was pursued to decrease motion artifacts between the two acquisition phases.

|  |  | mRECIST (patient based) | | | |
| --- | --- | --- | --- | --- | --- |
|  |  | ORR | CR | PR | PD |
| Visual degree of embolization (DE) | < 50 | 7/30 (23 %) | 6/30 (20 %) | 5/30 (17 %) | 1/30 (3 %) |
|  | > 50 | 12/30 (40 %) | 10/30 (33 %) | 6/30 (20 %) | 2/30 (7 %) |
|  | < 50 vs. > 50 | .3387 | .3226 | .7633 | .5714 |
| Embolic density (ED) | < 2 | 3/30 (10 %) | 2/30 (7 %) | 6/30 (20 %) | 1/30 (3 %) |
|  | ≥ 2 | 16/30 (53 %) | 14/30 (13 %) | 5/30 (17 %) | 2/30 (7 %) |
|  | < 2 vs. ≥ 2 | .0401* | .01* | .7633 | .5714 |
| Residual tumor perfusion (RTP) | yes | 4/30 (13 %) | 2/30 (7 %) | 9/30 (30 %) | 2/30 (7 %) |
|  | no | 15/30 (50 %) | 14/30 (13 %) | 2/30 (7 %) | 1/30 (3 %) |
|  | yes vs. no | .3387 | .01* | .0544 | .5714 |

**Supplemental Table 2.** Results of patient based analysis of 30 patients with 50 HCC lesions treated with rDEE-TACE according to visual degree of embolization (DE), embolic density (ED) and residual tumor perfusion (RTP). Most severe HCC lesion in each patient was taken into account when evaluating based on mRECIST (Modified Response Evaluation Criteria in Solid Tumors) criteria. ORR = objective response rate, CR = complete response, PR = partial response, PD = progressive disease.
